# Sternoclavicular Joint Arthritis in a Patient with Psoriatic Arthritis

**DOI:** 10.31138/mjr.290823.sja

**Published:** 2023-08-29

**Authors:** Takashi Nawata, Masafumi Yano

**Affiliations:** Department of Medicine and Clinical Science, Yamaguchi University Graduate School of Medicine, Ube, Japan

**Keywords:** sternoclavicular joint arthritis, psoriatic arthritis

## CASE PRESENTATION

**Figure 1.**
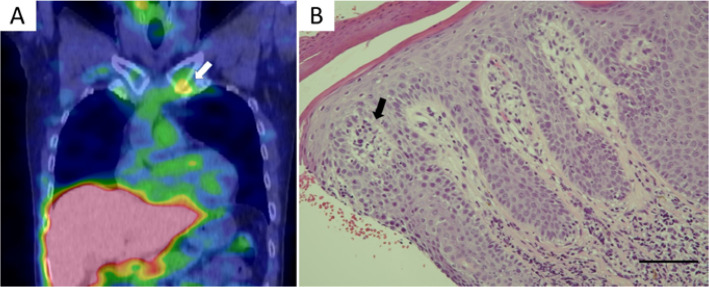
(A) Gallium scintigraphy revealed increased uptake in the left sternoclavicular joint. (B) Skin biopsy of the head erythema revealed parakeratosis and microabscess leading to the diagnosis of psoriatic arthritis (PsA).

A 45-year-old man presented with chronic left sterno-clavicular and bilateral fingers joint pain. In addition to polyarthralgia, he presented with fingernail depression and head erythema. Serum rheumatoid factor, anti-cyclic citrullinated peptide antibodies, and antinuclear antibodies were negative. Gallium scintigraphy revealed increased uptake in the left sternoclavicular joint (** Figure 1A**). Skin biopsy of the head erythema revealed parakeratosis and microabscess (**Figure 1B**) leading to the diagnosis of psoriatic arthritis (PsA). Non-steroidal anti-inflammatory drugs were started, but their effect was insufficient leading to the administration of adalimumab subcutaneously (80 mg biweekly). After administration of adalimumab, his skin and joint lesions improved.

## DISCUSSION

PsA occurs in patients with manifest or latent psoriasis. Axial involvement occurs in approximately 25 to 70% of patients with longstanding PsA, and in 5 to 28% of patients with early-stage disease.^1^ Sternoclavicular involvement is a rare manifestation of PsA. A study shows that two of 104 patients (1.9%) with PsA presented sternoclavicular joint arthritis.^2^ From the present case, psoriasis should be suspected in patients presenting sternoclavicular joint arthritis.
